# Endoscopic Diagnosis Strategy of Raspberry-Shaped Gastric Lesion in Helicobacter Pylori-Uninfected Patient

**DOI:** 10.3390/jcm12175437

**Published:** 2023-08-22

**Authors:** Nobuyuki Suzuki, Atsushi Ikeda, Hiroya Ueyama, Noboru Yatagai, Yasuko Uemura, Momoko Yamamoto, Tomoyo Iwano, Hisanori Utsunomiya, Ryota Uchida, Daiki Abe, Shotaro Oki, Yoichi Akazawa, Tsutomu Takeda, Kumiko Ueda, Mariko Hojo, Takashi Yao, Akihito Nagahara

**Affiliations:** 1 Department of Gastroenterology, School of Medicine, Juntendo University, Tokyo 113-8421, Japan; nb-suzuki@juntendo.ac.jp (N.S.);; 2 Department of Human Pathology, Graduate School of Medicine, Juntendo University, Tokyo 113-8421, Japan

**Keywords:** *Helicobacter pylori*-uninfected gastric cancer, gastric adenocarcinoma of foveolar type, raspberry-shaped gastric lesion, gastric adenocarcinoma of fundic-gland type, gastric adenocarcinoma of fundic-gland mucosa type, hyperplastic polyp, proton pump inhibitor-related lesion, early gastric cancer

## Abstract

Objectives: We aimed to clarify the endoscopic and clinicopathological features of raspberry-shaped gastric lesions (RSGLs) and to establish an endoscopic diagnostic algorithm for RSGLs. Methods: We collected RSGLs from an endoscopic database at our hospital between May 2009 and August 2021. All RSGLs were histopathologically classified and compared based on their endoscopic and clinicopathological characteristics. Results: Sixty-five RSGLs in 54 patients were classified into five histopathological types: gastric adenocarcinoma of foveolar type (GA-FV, *n* = 43), gastric adenocarcinoma of fundic-gland type (GA-FG, *n* = 2), gastric adenocarcinoma of fundic-gland mucosa type (GA-FGM, *n* = 4), hyperplastic polyp (HP, *n* = 12), and proton pump inhibitor-related lesion (PPI-L, *n* = 4). All RSGLs exhibited polygonal or curved marginal crypt epithelium (MCE). GA-FV lesions had homogenously reddish (95%) and an irregular microvascular (MV) pattern (91%). GA-FG lesions were heterogeneously reddish with a submucosal tumor shape (100%) and had a regular MV pattern (50%). GA-FGM lesions were homogen+ously reddish (75%) and occasionally had a submucosal tumor shape (50%) with an irregular MV pattern (75%). HPs and PPI-Ls were homogeneously reddish (93%), with linear or dotted MCE (81%) and a regular MV pattern (100%). Conclusion: Our diagnostic algorithm for RSGLs constructed using endoscopic features might be useful for the endoscopic differential diagnosis of RSGLs.

## 1. Introduction

Gastric cancer development presents with histological changes resulting from *Helicobacter pylori* (*H. pylori*) infection, such as atrophic gastritis and intestinal metaplasia [1,2]. In Japan, most patients with gastric cancer are *H. pylori*-positive or undergoing *H. pylori*-eradication therapy, and *H. pylori*-uninfected gastric cancer is considered rare (0.42–2.5% of gastric cancer) [3,4,5,6,7]. Recently, the proportion of *H. pylori*-positive patients has gradually decreased due to the widespread use of *H. pylori* eradication therapy; consequently, the incidence of gastric cancer without *H. pylori* infection has been gradually increasing [5].

Gastric adenocarcinoma of foveolar type (GA-FV), which is diagnosed as a differentiated type of gastric cancer with low-glade atypia in *H. pylori*-uninfected gastric cancers, has recently been categorized as a subtype of raspberry-shaped gastric cancers (RSGCs), which appear reddish protruded lesions with a fine granular surface [8,9,10,11,12]. According to the Japanese Classification of Gastric Carcinoma (JCGC), GA-FV is a well-differentiated adenocarcinoma. However, per the WHO criteria, it is a foveolar-type adenoma [9,10,13]. According to previous studies, there is a tendency for gastric adenocarcinoma of fundic-gland type (GA-FG) and gastric adenocarcinoma of fundic-gland mucosa type (GA-FGM) to differentiate into foveolar epithelial cells, fundic gland cells, or mucus neck cells [14,15]. In addition, some cases of GA-FG and GA-FGM present with reddish protruded lesions similar to those observed in RSGC. We previously reported the clinicopathological and endoscopic features of gastric cancers that resembled GA-FV in *H. pylori*-uninfected patients, and we subclassified RSGCs histopathologically into GA-FV, GA-FG, and GA-FGM [10]. However, benign lesions that resemble RSGCs are often observed in clinical practice. The classification, clinicopathological, and endoscopic features of raspberry-shaped benign gastric lesions (RSBGLs) have not been adequately elucidated.

Therefore, this study aimed to establish a new histopathological classification of raspberry-shaped gastric lesions (RSGLs), which include RSGC and RSBGL, and clarify endoscopic and clinicopathological features of each subtype of RSGLs in *H. pylori*-uninfected patients.

## 2. Materials and Methods

### 2.1. Study Design

We collected RSGCs in *H. pylori*-uninfected patients from 1048 early gastric cancer lesions resected using an endoscopic procedure and RSBGLs that endoscopically resembled RSGC at our hospital between April 2009 and August 2021. We designed a new histopathological classification of RSGLs and compared it using endoscopic and clinicopathological evaluations. In addition, we investigated the endoscopic findings that were useful for establishing a diagnostic algorithm for RSGLs.

### 2.2. H. pylori Infection Status

Absence of *H. pylori* infection was defined as per the following four criteria:

1. Lack of gastric mucosal atrophy in endoscopy or Kimura–Takemoto Classification of C-1 [16];
2. Absence of gastric atrophy in resected specimens as per pathological examinations;
3. Negative clinical findings observed for at least one of the following three tests: The 13C-urea breath test (UBT; cutoff value <2.5%, Otsuka, Tokushima, Japan), the *H. pylori* stool antigen test (Premier Platinum *H. pylori* SA; Meridian, Cincinnati, OH, USA), or lack of serum immunoglobulin G antibodies against *H. pylori* (*H. pylori* Ab; cutoff value < 3 U/mL, Eiken, Tokyo, Japan);
4. No history of previous therapy for *H. pylori* eradication [16,17].

### 2.3. Immunohistochemical Examination and Phenotypic Classification

Hematoxylin and eosin (H&E) staining and immunohistochemistry were performed on resected specimens after they were formalin-fixed and sectioned. The following markers were targeted with their respective monoclonal antibodies: MUC6 (Novocastra, Newcastle upon Tyne, UK) as a gastric mucous neck cell and pyloric gland marker, MUC5AC (Novocastra, Newcastle upon Tyne, UK) as a gastric foveolar cell marker, MUC2 (Leica, Newcastle upon Tyne, UK) as an intestinal goblet cell marker, CD10 (Novocastra, Newcastle upon Tyne, UK) as a small intestinal brush border marker, H^+^/K^+^-ATPase subunit (MBL, Nagoya, Japan) as a parietal cell marker, pepsinogen-I (Abcam, Cambridge, UK) as a chief cell marker, p53 (BioGenex, Fremont, CA, USA), and Ki-67 (MIB-1, DAKO, Glostrup, Denmark). Positivity was defined as >10% of the sample area exhibiting staining. The percentage of MIB-1-positive nuclei was used as the Ki-67 MIB-1 labeling index (LI), which was evaluated by observing at least 1000 nuclei in selected fields at ×400 magnification. In addition, gastric mucin (MUC5AC and MUC6) and intestinal mucin (MUC2, CD10) phenotypes were determined using immunohistochemistry (Figure S1).

### 2.4. Histopathological Classification of RSGL

**Figure 1:**
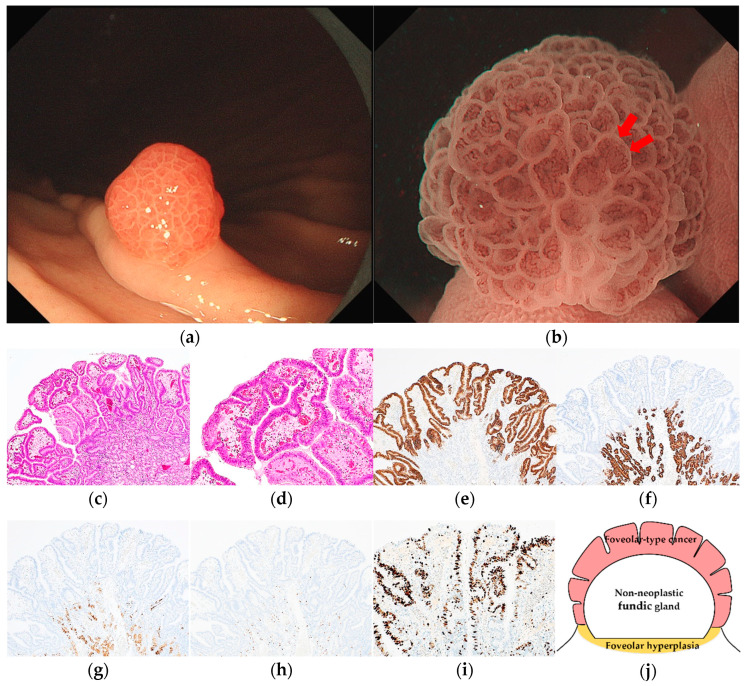
Endoscopic and pathological findings of GA-FV. (**a**) WLI reveals a reddish superficial elevated lesion, with an approximate size of 5 mm, on the greater curvature of the upper third of the stomach. The MCE shape is only polygonal or curved; (**b**) ME-NBI shows an irregular MV pattern plus a regular MS pattern with a demarcation line and an irregular inner edge shape of MCE (red arrow); (**c**,**d**) Hematoxylin and eosin (H&E) stain. Histological findings of endoscopic mucosal resection specimen indicate well-differentiated adenocarcinoma that mimicked foveolar epithelium in the superficial layer; (**e**–**i**) immunostaining; (**e**) MUC5AC (gastric foveolar epithelial cell). Diffusely positive; (**f**) MUC6 (gastric mucous neck cell). Negative; (**g**) pepsinogen-I (chief cell). Negative; (**h**) H^+^/K^+^-ATPase (parietal cell). Negative; (**i**) Ki-67 is overexpressed (Labeling index 80%); (**j**) the structural schema of GA-FV is presented. Original magnification: (**c**,**e**–**h**) ×40, (**d**,**i**) ×100. [M, Less, 0-I, 5 × 4 mm, pap, pT1a/M, pUL0, Ly0, V0, pHM0, pVM0].

**Figure 2:**
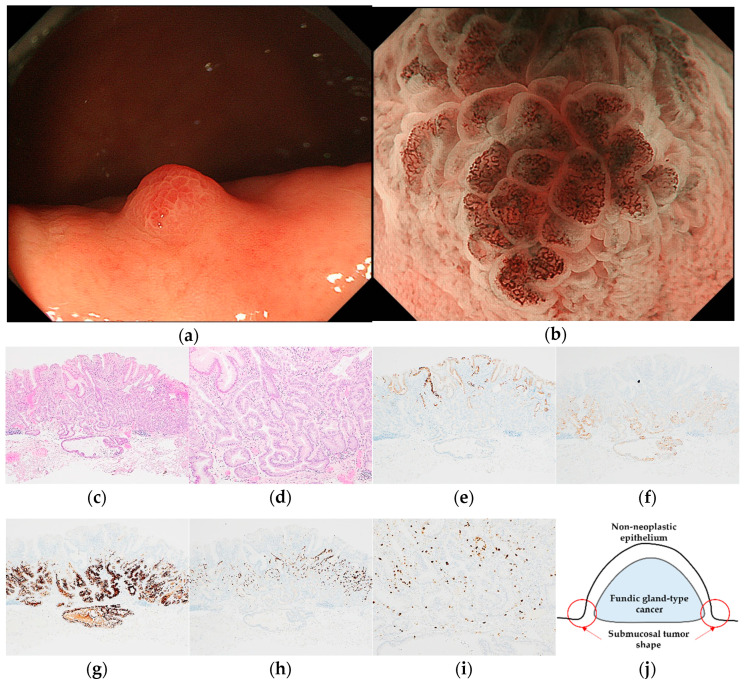
Endoscopic and pathological findings of GA-FG. (**a**) WLI reveals a heterogenous reddish protruded lesion with a submucosal tumor shape, with an approximate size of 5 mm, on the greater curvature of the upper third of the stomach; (**b**) ME-NBI shows an irregular MV pattern plus a regular MS pattern with a demarcation line; (**c**,**d**) H&E stain. Histological findings of the endoscopic submucosal dissection specimen indicate well-differentiated adenocarcinoma that mimicked gastric fundic glands structured as irregular branches in the deep layer of the lamina propria mucosa, infiltrating the submucosal layer (300 μm). The superficial layer of the tumor margin is covered with a non-neoplastic epithelium; (**e**–**i**) immunostaining; (**e**) MUC5AC (gastric foveolar epithelial cell). Negative; (**f**) MUC6 (gastric mucous neck cell). Positive; (**g**) pepsinogen-I (chief cell). Diffusely positive; (**h**) H^+^/K^+^-ATPase (parietal cell). Positive; (**i**) Ki-67 is overexpressed (Labeling index 10%); (**j**) the structural schema of GA-FG is presented. Original magnification: (**c**,**e**–**h**) ×40, (**d**,**i**) ×100. [U, Gre, 0-I, 5 × 4 mm, adenocarcinoma of fundic gland type, pT1b1/SM1 (300 μm), pUL0, Ly0, V0, pHM0, pVM0].

**Figure 3:**
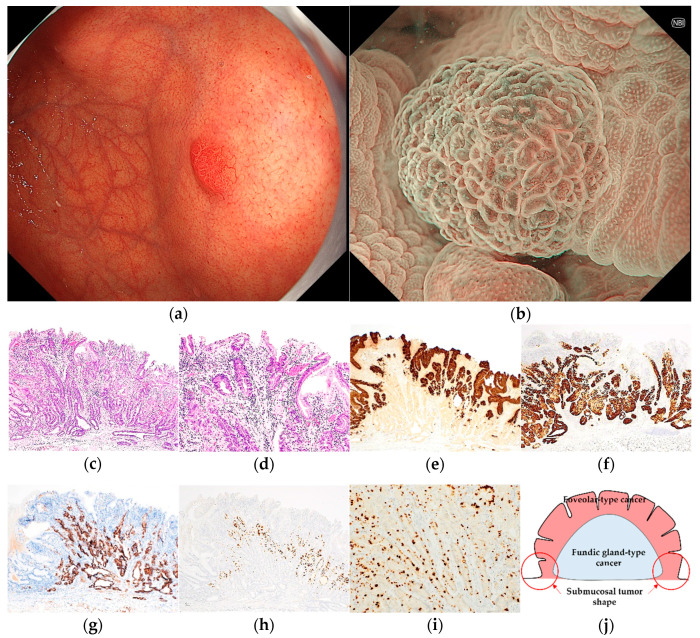
Endoscopic and pathological findings of GA-FGM. (**a**) WLI reveals a homogenous reddish protruded lesion with a submucosal tumor shape, with an approximate size of 8 mm, on the anterior wall of the upper third of the stomach. The MCE shape is both polygonal/curved and linear/dotted; (**b**) ME-NBI shows a regular MV pattern plus an irregular MS pattern with a demarcation line; (**c**,**d**) H&E stain. Histological findings of the endoscopic submucosal dissection specimen indicate well-differentiated adenocarcinoma that mimicked foveolar epithelium in the superficial layer and mimicked gastric fundic glands structured as irregular branches in the deep layer of the lamina propria mucosa, which infiltrated the submucosal layer (300 μm); (**e**–**i**) immunostaining; (**e**) MUC5AC (gastric foveolar epithelial cell). Positive; (**f**) MUC6 (gastric mucous neck cell). Positive; (**g**) pepsinogen-I (chief cell). Diffusely positive; (**h**) H^+^/K^+^-ATPase (parietal cell). Positive; (**i**) Ki-67 is overexpressed (Labeling index 20%); (**j**) the structural schema of GA-FGM is presented. Original magnification: (**c**,**e**–**h**) ×40, (**d**,**i**) ×100. [U, Gre, 0-I, 8 × 7 mm, tub1, pT1b1/SM1 (300 μm), INFa, pUL0, Ly0, V0, pHM0, pVM0].

Pathological diagnoses of gastric cancer were defined as follows: GA-FV was indicated by differentiated adenocarcinoma comprising cells resembling foveolar epithelium and positivity of MUC5AC, a marker for gastric foveolar cells (Figure 1c–i); GA-FG was indicated by differentiated adenocarcinoma consisting of cells resembling fundic glands and pepsinogen-I and/or H^+^/K^+^-ATPase positivity (Figure 2c–i); GA-FGM was indicated by differentiated adenocarcinoma comprising cells resembling the foveolar epithelium and fundic glands (Figure 3c–i). Additionally, MUC5AC positivity was required for neoplastic foveolar epithelium, and pepsinogen-I and/or H^+^/K^+^-ATPase positivity was required for neoplastic fundic gland cells. The pathological diagnoses of proton pump inhibitor-related lesion (PPI-L) were made using the following criteria: dilation and elongation of the foveolar epithelium, fundic gland polyps with parietal cell hyperplasia and protrusion, and vascularity of stroma associated with long-term use of PPI [18,19,20]. A gastrointestinal pathology specialist (T.Y.) performed all histopathological diagnoses.

### 2.5. Clinicopathological Assessment

Clinicopathological findings included age, sex, PPI administration, smoking history, alcohol history, survival period, outcome, tumor location, tumor size, therapy method, depth of invasion, mean depth of submucosal invasion, presence of lymphatic and vascular invasion, lateral and vertical margins, presence of foveolar hyperplasia around the tumor, and presence of covered or mixed non-neoplastic epithelium. The survival period was defined as the survival time from treatment to the last outpatient visit or annual endoscopy.

### 2.6. Endoscopic Assessment

A raspberry-like reddish protruded lesion with white-light imaging (WLI) was diagnosed as an RSGL. Endoscopic findings with WLI included redness (homogeneous/heterogeneous), the type of shape of the marginal crypt epithelium (MCE) (polygonal, curved, linear, or dotted), submucosal tumor shape, the presence of a whitish area around the tumor, the coexistence of multiple white and flat elevated lesions, and fundic gland polyps.

To diagnose early gastric cancer with magnifying endoscopy with narrow-band imaging (ME-NBI), a “vessel plus surface” classification system [21], the main algorithm for magnifying endoscopy simple diagnostic algorithm for early gastric cancer (MESDA-G) [22], was used. In the analysis of endoscopic findings with ME-NBI, the following factors were considered: the presence of a demarcation line, microvascular (MV) pattern, microsurface (MS) pattern, and irregular inner edge shape of the MCE (Figure 2b). All WLI and ME-NBI images were retrospectively analyzed by three expert endoscopists (N.S., A.I., and H.U.). In the case of different diagnoses, a consensus was reached following a discussion between these experts.

## 3. Results

Sixty-five RSGLs from 54 patients were histologically classified as RSGCs (*n* = 49) and RSBGLs (*n* = 16). Forty-nine RSGCs were subclassified into 43 GA-FV lesions, 2 GA-FG lesions, and 4 GA-FGM lesions. Sixteen RSBGLs were subclassified into 12 hyperplastic polyps (HPs) and 4 PPI-Ls. One PPI-L contained low-grade dysplasia in part of the lesion. Three patients had 2 GA-FV lesions, 2 patients had 1 GA-FV lesion and 1 HP, 1 patient had 2 GA-FV lesions and 1 HP, and 1 patient had 4 GA-FV lesions and 1 HP.

In addition, 24 lesions of RSGCs (19 GA-FV, 2 GA-FG, 3 GA-FGM lesions) were the same as those analyzed in our previous report [10]. However, we report the results of this study as novel findings since our previous report did not include an analysis of RSBGLs, the shape of MCE in RSGCs, and the diagnostic algorithm of RSGLs.

### 3.1. Clinicopathological Features of RSGLs

The clinicopathological features of the RSGLs are summarized in Table 1. GA-FV occurred more frequently in males (27/36, 75.0%). The RSGCs were predominantly located in the greater curvature (44/49, 89.8%) of the upper or middle third of the stomach (49/49, 100%). HPs were more frequently located in the upper third of the stomach, especially in the cardia (4/12, 33.3%). PPI-Ls were more frequently located in the lower third of the stomach (2/4, 50.0%). The mean tumor size of the GA-FV lesions was 3.3 mm, which was smaller than that of other subtypes of RSGLs. All GA-FV lesions remained in the mucosal layer. Two GA-FG lesions (2/2, 100%) and three GA-FGM lesions (3/4, 75.0%) showed invasion into the submucosal layer. None of the RSGCs showed lymphovascular invasion or lateral or vertical margins. One PPI-L lesion (1/4, 25.0%) showed a mix of atypical foveolar hyperplasia. The therapy methods were biopsy, cold forceps polypectomy (CFP), endoscopic mucosal resection (EMR), and endoscopic submucosal dissection (ESD) in 5/18/21/5 RSGCs and 6/7/3/0 RSBGLs. Foveolar hyperplasia around the tumor was observed in all GA-FV and 3 GA-FGM lesions (3/4, 75.0%). Covered or mixed non-neoplastic epithelium of the tumor showed 13 GA-FV lesions (13/41, 31.7%), 2 GA-FG lesions (2/2, 100%), and 1 GA-FGM lesion (1/4, 25.0%). Foveolar hyperplasia around the tumor and covered mixed non-neoplastic epithelium were unevaluated in 2 GA-FV lesions.

### 3.2. Immunohistochemical Features of RSGLs

The immunohistochemical features of RSGLs are summarized in Table 2. The immunohistochemical features of all RSGCs were the same as those previously reported [10]. All RSGLs were positive for MAC5AC and/or MUC6 and were classified as the gastric mucin phenotype. The mean Ki-67 MIB-1 LI of GA-FV was relatively higher than that of the other RSGCs (GA-FV/GA-FG/GA-FGM: 43/15/23%). No RSGC exhibited p53 overexpression.

### 3.3. Endoscopic Features of RSGLs

The endoscopic features of RSGLs on WLI and ME-NBI are summarized in Table 3. All RSGLs included polygonal or curved shapes of the MCE in the WLI. GA-FV was characterized by homogenous reddish lesions (41/43, 95.3%) and was frequently accompanied by only polygonal or curved MCE (40/43, 93.0%) and a whitish area around the tumor (30/43, 69.8%) in WLI. No GA-FV lesions showed submucosal tumor shape (0/43, 0%). Most GA-FV lesions were diagnosed as cancer by an irregular MV pattern or irregular inner edge of the MCE in ME-NBI (30/33, 90.9%) (Figure 1).

GA-FG was characterized by a heterogeneous reddish submucosal tumor shape (2/2, 100%) (Figure 2). Meanwhile, GA-FGM was characterized by homogenous reddish (3/4, 75.0%) with mixed linear or dotted shape of MCE (3/4, 75.0%) and sometimes accompanied by submucosal tumor shape (2/4, 50.0%) in WLI. Most GA-FGM lesions were diagnosed as cancer by irregular MV pattern (3/4, 75.0%) or irregular inner edge of the MCE (4/4, 100%) in ME-NBI (Figure 3).

**Figure 4:**
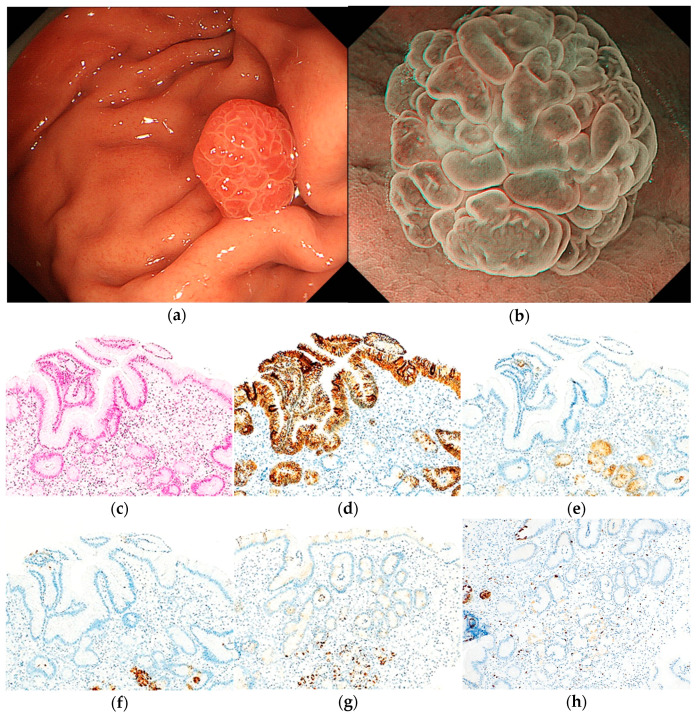
Endoscopic and pathological findings of HP. (**a**) WLI reveals a homogenous reddish protruded lesion, with an approximate size of 6 mm, on the greater curvature of the upper third of the stomach. The MCE shape is both polygonal/curved and linear/dotted; (**b**) ME-NBI shows an absent MV pattern plus a regular MS pattern with a demarcation line; (**c**) H&E stain. Histological findings of the biopsy specimen indicate foveolar epithelial hyperplasia with cystic dilatations and edema and inflammation of lamina propria; (**d**–**h**) immunostaining; (**d**) MUC5AC (gastric foveolar epithelial cell). Positive; (**e**) MUC6 (gastric mucous neck cell). Negative; (**f**) pepsinogen-I (chief cell). Negative; (**g**) H^+^/K^+^-ATPase (parietal cell). Negative; (**h**) Ki-67 is not overexpressed. Original magnification: (**c**–**h**) ×100.

**Figure 5:**
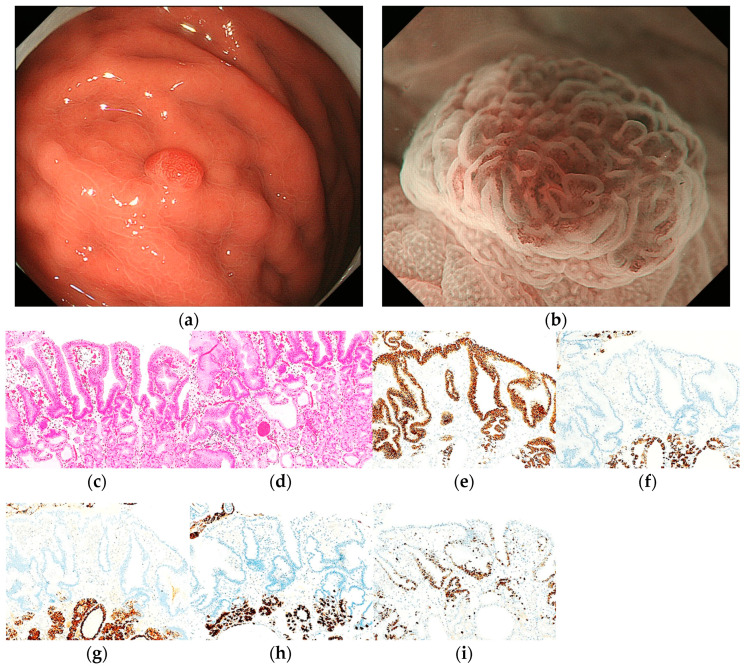
Endoscopic and pathological findings of PPI-L with low-grade dysplasia. (**a**) WLI reveals a homogenous reddish protruded lesion, with an approximate size of 6 mm, on the greater curvature of the upper third of the stomach. The MCE shape is both polygonal/curved and linear/dotted; (**b**) ME-NBI shows a regular MV pattern plus a regular MS pattern with a demarcation line; (**c**,**d**) H&E stain. Histological findings of the CFP specimen indicate parietal cell hyperplasia and protrusion, dilation and elongation of the foveolar epithelium, and vascularity of stroma. The surface of the PPI-L shows low-grade dysplasia; (**e**–**i**) immunostaining; (**e**) MUC5AC (gastric foveolar epithelial cell). Positive; (**f**) MUC6 (gastric mucous neck cell). Negative; (**g**) pepsinogen-I (chief cell). Negative; (**h**) H^+^/K^+^-ATPase (parietal cell). Negative; (**i**) Ki-67 is overexpressed (Labeling index 80%). Original magnification: (**c**–**i**) ×100.

RSBGLs were characterized by homogenous reddish (15/16, 93.8%) and mixed linear or dotted shape of MCE (13/16, 81.3%) without submucosal tumor shape (0/16, 0%) in WLI. All RSBGLs were diagnosed as non-cancerous using MESDA-G in ME-NBI (Figure 4 and Figure 5).

**Figure 6:**
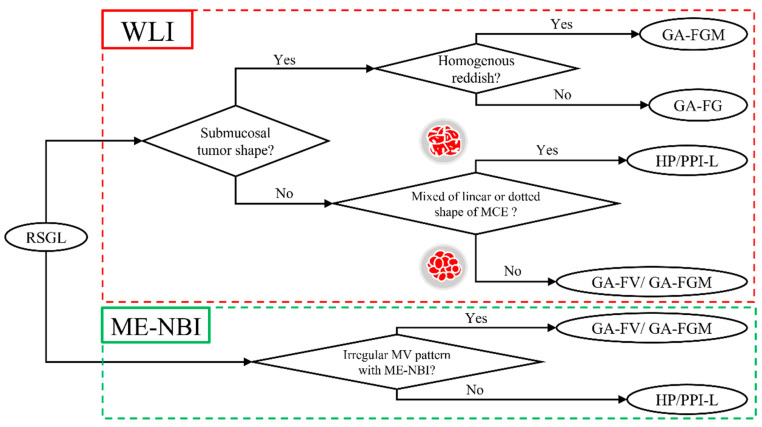
Diagnostic algorithm of RSGL.

We established a diagnostic algorithm for RSGLs based on the following endoscopic features (Figure 6). If RSGL is detected, the first step is to identify the presence of a submucosal tumor shape in WLI. If a submucosal tumor shape is present, the next step is to evaluate the redness of the lesion using WLI. If the redness of the lesion is homogenous, GA-FGM may be diagnosed; if the redness of the lesion is heterogeneous, GA-FG may be diagnosed. If the submucosal tumor shape is absent, the second step is to evaluate the mixed linear or dotted shape of the MCE using WLI. If there is a mixed linear or dotted shape of the MCE, RSBGL may be diagnosed; if there is no mixed linear or dotted shape of MCE, GA-FV/GA-FGM may be diagnosed. According to ME-NBI, if the MV is irregular, a diagnosis of GA-FV/GA-FGM may be made; if the MV is regular/absent, RSBGL may be diagnosed.

## 4. Discussion

As a result of the 65 RSGLs analyzed, we clarified the endoscopic and clinicopathological features and established a diagnostic algorithm for RSGLs. This study is the first report of endoscopic differentiation of RSGLs in *H. pylori*-uninfected patients using WLI and ME-NBI. 

Herein, the frequency of RSGCs in patients without *H. pylori* infection (49/1048, 4.7%) was higher than that in patients with *H. pylori* infection in previous reports [3,4]. It is possible that in past studies using endoscopic evaluations, RSGCs may have been overlooked because endoscopists may have originally considered that no cancer occurred in the *H. pylori*-uninfected stomach and diagnosed reddish protruded lesions in *H. pylori*-uninfected stomach as HP. As the rate of cancer is high (49/65, 75.4%) in RSGLs, endoscopists should recognize the possibility of RSGCs when they detect RSGLs in upper gastrointestinal endoscopy. When RSGLs are detected, a differentiated diagnosis of RSGLs can be made using a diagnostic algorithm that comprises the presence of a submucosal tumor shape, the type of reddish coloration, and the shape of the MCE in WLI. In addition, the MV pattern on ME-NBI is useful for the differential diagnosis between RSGCs and RSBGLs. 

As for endoscopic and histopathologic findings for each subtype of RSGL, GA-FG, and GA-FGM appear as submucosal tumor shapes endoscopically because GA-FG (2/2, 100%) and GA-FGM (2/4, 50.0%) tumor cells exist in the deep part of the mucosal layer histologically [23]. GA-FG shows heterogeneous redness in WLI and a regular MV pattern in ME-NBI endoscopically because tumor cells of GA-FG are covered with non-neoplastic foveolar epithelium histologically [23]. GA-FV and GA-FGM exhibit homogenous redness in WLI and an irregular MV pattern in ME-NBI because their tumor cells are histologically exposed on the surface of the epithelium [24,25,26]. In most lesions of GA-FV and GA-FGM, the shape of MCE is only polygonal or curved in WLI because the tumor cells of GA-FV and GA-FGM grow monotonously without non-neoplastic gastric mucosa histologically. In most RSBGLs, the shape of the MCE is not only polygonal or curved but also linear or dotted in WLI because the cells of RSBGLs grow to mimic the gastric oxyntic gland, which contains linear or dotted shaped MCE histologically. Therefore, it was suggested that RSGLs with a mixed linear or dotted MCE shape are likely to be RSBGLs. All RSBGLs appear as regular MV patterns in ME-NBI because they are not cancerous. If tumor cells are histologically exposed on the surface of the epithelium, we can diagnose the lesion as cancer by the endoscopic finding of a change in the regular MV/MS pattern to an irregular MV/MS pattern. This finding is important for the differential diagnosis of RSGLs. A diagnostic algorithm based only on WLI findings is useful because of the high positive diagnosis rate (57/65, 87.7%), and combined with ME-NBI findings, the positive diagnosis rate further increased. 

Similar to previous studies, all RSGCs were located in the upper or middle third of the stomach and the greater curvature or anterior/posterior wall [10,14]. Moreover, 3 and 7 RSBGLs were located in the lower third of the stomach (3/16, 18.8%) and in the lesser curvature (7/16, 43.8%), respectively. Therefore, RSGLs located in the lower third of the stomach or the lesser curvature are likely to be RSBGLs. In the case of RSGLs that are difficult to differentiate using this algorithm, the location of the lesion may help differentiate RSGC and RSBGL. 

In our previous report, the presence of a whitish area around the tumor, one of the endoscopic features of GA-FV in WLI, was useful for the endoscopic differentiation of RSGCs [10]; similarly, it was observed frequently in this present study (30/43, 70%). However, we did not include it in the algorithm because five RSBGLs appeared as a whitish area around the tumor (5/16, 31%). The irregular inner edge shape of the MCE is frequently observed in RSGCs (35/39, 89.7%) and may be useful for endoscopic differentiation [10]. Nevertheless, we did not include it in the algorithm because the irregularity of the inner edge of the MCE was due to the deformation of the MV outer edge. We consider that the identification of the MV pattern is more important than the irregularity of the inner edge of the MCE. Therefore, we included the MV pattern in the diagnostic algorithm instead of the inner edge shape of the MCE.

RSGCs were diagnosed as cancer by biopsy and were treated endoscopically. However, the pathological differential diagnosis between GA-FV and regenerative atypia by biopsy is sometimes difficult and may require the diagnosis of expert gastrointestinal pathologists [9,12,27]. In the case of a biopsy of small lesions (especially GA-FV), less than 5 mm, the lesion often disappears, and only a scar remains at the next endoscopy despite the lesion remaining just after the biopsy, and it is impossible to evaluate whether curative resection has been achieved pathologically. When we found a lesion of suspected GA-FV by the diagnostic algorithm less than 5 mm in size, we performed en bloc resection using CFP, cold snare polypectomy (CSP), or EMR without biopsy after fully obtaining informed consent, and we histopathologically evaluated whether a curative resection had been achieved. Consequently, all GA-FV lesions, less than 5 mm in size, resected by EMR, CFP, and CSP, achieved curative resection. Although further analysis of prognosis and recurrence is needed, CFP and CSP may be treatment options for small GA-FV lesions. In cases of GA-FV lesions >5 mm, EMR should be performed to achieve curative resection. If the lesion is suspected to be GA-FG/GA-FGM, en bloc resection by EMR/ESD was considered preferable to achieve a negative vertical margin because GA-FG/GA-FGM lesions tend to invade the submucosal layer regardless of their size. If RSGLs are diagnosed as RSBGLs using the algorithm, the lesion does not require endoscopic therapy. If endoscopic differentiation of RSBGLs from RSGCs is difficult, a pathological diagnosis should be made via biopsy.

The immunohistochemical features of the RSGCs were the same as those previously reported. Although we have reported that GA-FV lesions are smaller than that of GA-FG or GA-FGM despite higher Ki-67 MIB-1 LI, indicating proliferative potential, the reason for this has not been elucidated [10]. Mishiro et al. reported that single-nucleotide variations in KLF4, associated with apoptosis, were present in GA-FV lesions and may be responsible for the high Ki-67 LI [28].

This study had some limitations. First, this study was a retrospective observational analysis performed at a single center with a small sample size. Second, the diagnostic algorithm was not validated in a prospective study. Therefore, a multicenter prospective study is needed to validate the diagnostic algorithm in the future. Third, our report contained the clinicopathological data of 24 lesions from a previous study [10]. However, the endoscopic features and endoscopic differentiation of RSGLs have not been thoroughly investigated. Thus, the fact that some lesions overlap with the previous report does not undermine the novelty of this study. In addition, this diagnostic algorithm may seem to require specialized knowledge and training. However, we believe this diagnostic algorithm can be used by beginners or non-specialized centers since the endoscopic findings used in this algorithm are relatively easy to visualize and judge in WLI.

## 5. Conclusions

RSGLs in *H. pylori*-uninfected patients were histopathologically classified as GA-FV, GA-FG, GA-FGM, HP, and PPI-L, and we established a new diagnostic algorithm based on the endoscopic features of each subtype. This study suggests this new diagnostic algorithm might be useful for endoscopic differentiation and treatment strategy determination for RSGLs.

## Figures and Tables

**Table 1:** Clinicopathological features of RSGLs.

	RSGC			RSBGL	
	GA-FV	GA-FG	GA-FGM	HP	PPI-L
Patients (*n* = 54)	*n* = 36	*n* = 2	*n* = 4	*n* = 12	*n* = 4
Age (mean), years (range)	56.1 (29–81)	65.5 (51–80)	51.3 (40–63)	65.5 (46–76)	67.8 (54–81)
Sex (male/female)	27/9	0/2	2/2	6/6	2/2
PPI administration (+/−)	9/27	0/2	0/4	4/8	4/0
Smoking history (+/−)	22/14	1/1	2/2	6/6	1/3
Alcoholic history (+/−)	13/23	1/1	1/3	3/9	0/4
Survival periods (mean), months (range)	26.2 (1–141)	77.0 (45–109)	24.3 (1–76)	NA	NA
Outcome	One death due to another disease	All alive NED	All alive NED	NA	NA
Lesions (*n* = 65)	*n* = 43	*n* = 2	*n* = 4	*n* = 12	*n* = 4
Endoscopic findings					
Location (U/M/L)	27/16/0	2/0/0	4/0/0	7/4/1	1/1/2
(GC/LC/Ant/Post)	42/0/0/1	2/0/0/0	0/0/2/2	7/5/0/0	2/2/0/0
Size (average), mm	3.3 (2–6)	8.5 (5–12)	8.0 (4–15)	4.8 (1–15)	8.5 (3–20)
Therapy method (biopsy/CFP/EMR/ESD)	5/18/20/0	0/0/0/2	0/0/1/3	4/6/2/0	2/1/1/0
Pathological findings					
Invasion depth (M/SM)	43/0	0/2	1/3	NA	NA
Distance of SM invasion (average), μm (range)	NA	250 (200–300)	433 (300–700)	NA	NA
Lymphatic invasion (+/−)	0/43	0/2	0/4	NA	NA
Venous invasion (+/−)	0/43	0/2	0/4	NA	NA
Lateral margin (+/−)	0/43	0/2	0/4	NA	NA
Vertical margin (+/−)	0/43	0/2	0/4	NA	NA
Foveolar hyperplasia around tumor (+/−)	41/0	0/2	3/1	NA	NA
Covered or mixed non-neoplastic epithelium (+/−)	13/28	2/0	1/3	NA	NA

RSGC—raspberry-shaped gastric cancer; RSBGL—raspberry-shaped benign gastric lesion; GA-FV—gastric adenocarcinoma of foveolar type; GA-FG—gastric adenocarcinoma of fundic-gland type; GA-FGM—gastric adenocarcinoma of fundic-gland mucosa type; HP—hyperplastic polyp; PPI-L—proton pump inhibitor-related lesion; U—upper third; M—middle third; L—lower third; GC—greater curvature; LC—lesser curvature; Ant—anterior wall; Post—posterior wall; CFP—cold forceps polypectomy; EMR—endoscopic mucosal resection; ESD—endoscopic submucosal dissection; M—mucosal layer; SM—submucosal layer; NA—not assessed; NED—no evidence of disease.

**Table 2:** Immunohistochemical features of RSGLs.

	RSGC			RSBGL	
	GA-FV	GA-FG	GA-FGM	HP	PPI-L
Lesions	*n* = 40	*n* = 2	*n* = 4	*n* = 9	*n* = 4
MUC5AC (+/−)	40/0	0/2	4/0	9/0	4/0
MUC6 (+/−)	0/40	2/0	4/0	9/0	3/1
MUC2 (+/−)	0/40	0/2	0/4	0/9	0/4
CD10 (+/−)	0/40	0/2	0/4	0/9	0/4
Gastric phenotype (+/−)	40/0	2/0	4/0	9/0	4/0
pepsinogen-I (+/−)	0/40	2/0	4/0	4/5	4/0
H^+^/K^+^-ATPase (+/−)	0/40	2/0	4/0	3/6	4/0
Ki-67 MIB-1 labeling index (%) (mean)	42.8	15.0	22.5	NA	NA
(range)	5–80	10–20	10–30	NA	NA
p53 over expression (+/−)	0/40	0/2	0/4	NA	NA

RSGC—raspberry-shaped gastric cancer; RSBGL—raspberry-shaped benign gastric lesion; GA-FV—gastric adenocarcinoma of foveolar type; GA-FG—gastric adenocarcinoma of fundic-gland type; GA-FGM—gastric adenocarcinoma of fundic-gland mucosa type; HP—hyperplastic polyp; PPI-L—proton pump inhibitor-related lesion.

**Table 3:** Endoscopic features of RSGLs.

	RSGC			RSBGL	
	GA-FV	GA-FG	GA-FGM	HP	PPI-L
WLI	*n* = 43	*n* = 2	*n* = 4	*n* = 12	*n* = 4
Redness (homogenous/heterogeneous)	41/2	0/2	3/1	11/1	4/0
Shape of MCE (polygonal or curved) (+/−)	43/0	2/0	4/0	12/0	4/0
Shape of MCE (linear or dotted) (+/−)	3/40	1/1	3/1	9/3	4/0
Whitish area around tumor (+/−)	30/13	0/2	0/4	4/8	1/3
Submucosal tumor shape (+/−)	0/43	2/0	2/2	0/12	0/4
Multiple white and flat elevated lesions (+/−)	5/38	0/2	0/4	2/10	1/3
Fundic gland polyp (+/−)	33/10	2/0	1/3	9/3	4/0
ME-NBI	*n* = 33	*n* = 2	*n* = 4	*n* = 11	*n* = 3
MESDA-G (cancer/non-cancer)	30/3	1/1	3/1	0/11	0/3
Demarcation line (+/−)	33/0	2/0	4/0	11/0	3/0
Microvascular pattern (irregular/regular/absent)	30/0/3	1/1/0	3/0/1	0/9/2	0/2/1
Microsurface pattern (irregular/regular/absent)	0/33/0	0/2/0	0/4/0	0/11/0	0/3/0
Irregular inner edge shape of MCE (+/−)	30/3	1/1	4/0	0/11	0/3

RSGC—raspberry-shaped gastric cancer; RSBGL—raspberry-shaped benign gastric lesion; GA-FV—gastric adenocarcinoma of foveolar type; GA-FG—gastric adenocarcinoma of fundic-gland type; GA-FGM—gastric adenocarcinoma of fundic-gland mucosa type; HP—hyperplastic polyp; PPI-L—proton pump inhibitor-related lesion; WLI—white-light imaging; ME-NBI—magnifying endoscopy with narrow-band imaging; MESDA-G—magnifying endoscopy simple diagnostic algorithm for early gastric cancer.

## Data Availability

Not applicable.

## Supplementary Materials

The following supporting information can be downloaded at: https://www.mdpi.com/article/10.3390/jcm12175437/s1, Figure S1: Phenotypic classification.
