# Supplementary material for: Endoscopic Diagnosis Strategy of Raspberry-Shaped Gastric Lesion in Helicobacter Pylori-Uninfected Patient
Source: J Clin Med. 2023 Aug 22;12(17):5437. doi: 10.3390/jcm12175437 (PMC10488238; doi:10.3390/jcm12175437)
Supplement: Supplementary file 1 [file jcm-12-05437-s001.zip › supplementary figure.pptx]

## Slide 1
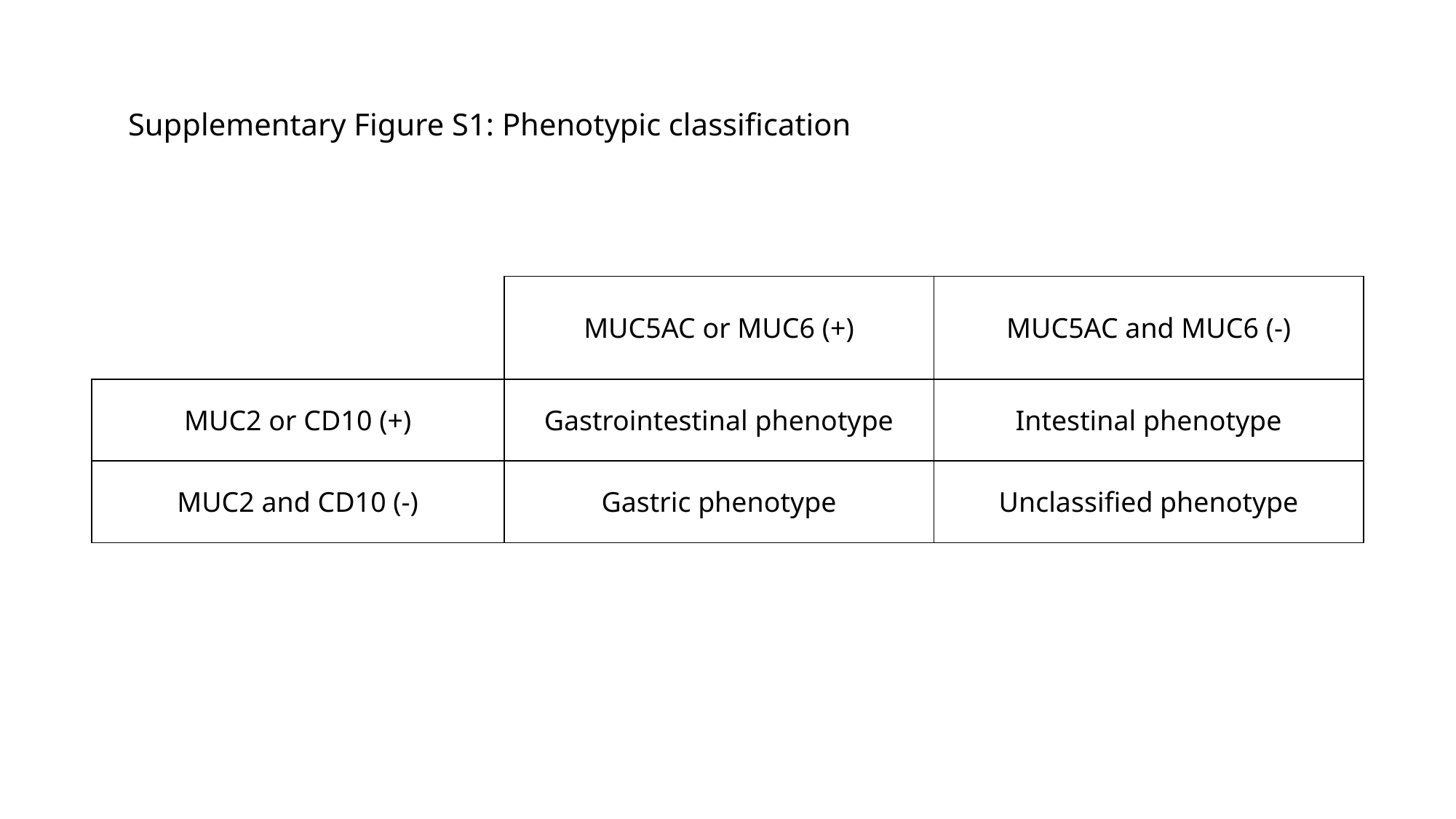

Supplementary Figure S1: Phenotypic classification
| | MUC5AC or MUC6 (+) | MUC5AC and MUC6 (-) |
| --- | --- | --- |
| MUC2 or CD10 (+) | Gastrointestinal phenotype | Intestinal phenotype |
| MUC2 and CD10 (-) | Gastric phenotype | Unclassified phenotype |
